# Experience of South and Southeast Asian minority women in Hong Kong during COVID-19 pandemic: a qualitative study

**DOI:** 10.1186/s12939-023-01922-6

**Published:** 2023-06-02

**Authors:** Roger Yat-Nork Chung, Tobey Tsz-Yan Lee, Siu-Ming Chan, Gary Ka-Ki Chung, Yat-Hang Chan, Samuel Yeung-Shan Wong, Eric Lai, Hung Wong, Eng Kiong Yeoh, Michael Marmot, Jean Woo

**Affiliations:** 1 CUHK Institute of Health Equity, The Chinese University of Hong Kong, Hong Kong, China; 2 JC School of Public Health and Primary Care, The Chinese University of Hong Kong, Hong Kong, China; 3 CUHK Centre for Bioethics, The Chinese University of Hong Kong, Hong Kong, China; 4 CUHK Institute of Ageing, The Chinese University of Hong Kong, Hong Kong, China; 5 Department of Social and Behavioural Sciences, City University of Hong Kong, Hong Kong, China; 6 Present Address: Department of Social Work, The Chinese University of Hong Kong, Hong Kong, China; 7 Department of Epidemiology and Public Health, UCL Institute of Health Equity, University College London, London, United Kingdom

**Keywords:** Ethnic minority, South Asians, Southeast Asians, Women, COVID-19, Health, Low incidence rate, Hong Kong

## Abstract

**Background:**

Hong Kong has a relatively low incidence rate of COVID-19 across the globe. Nevertheless, ethnic minorities in Hong Kong, especially South Asians (SAs) and Southeast Asians (SEAs), face numerous physical, mental, social, economic, cultural and religious challenges during the pandemic. This study explores the experiences of SA and SEA women in a predominantly Chinese metropolitan city.

**Methods:**

Ten SA and SEA women were recruited and face-to-face interviews were conducted. Questions about participants’ daily life experience, physical and mental health conditions, economic situation and social interaction amid COVID-19 pandemic were asked to assess the impact of COVID-19.

**Results:**

SAs and SEAs have a distinctive family culture, and women experienced significant physical and mental impact of COVID-19 due to their unique gender role in the family. In addition to taking care of their family in Hong Kong, SA and SEA women also had to mentally and financially support family members residing in their home countries. Access to COVID-related information was restricted due to language barrier. Public health measures including social distancing imposed extra burden on ethnic minorities with limited social and religious support.

**Conclusions:**

Even when COVID-19 incidence rate is relatively low in Hong Kong, the pandemic made life even more challenging for SAs and SEAs, which is a community already struggling with language barriers, financial woes, and discrimination. This in turn could have led to greater health inequalities. Government and civil organizations should take the social determinants of health inequalities into account when implementing COVID-19-related public health policies and strategies.

## Background

### Ethnic minorities situation during COVID-19 pandemic in the world

Coronavirus was thought to be non-discriminative initially as it threatened everyone in different regions across the globe. However, growing number of studies showed that COVID-19 pandemic has magnified the effect of health disparities [1, 2]. Studies in the countries with high infection rates showed that the death rates and infection rates of ethnic minorities were much higher than those of the general populations [3, 4]. The realistic situation faced by ethnic minorities may be worse since the related deaths maybe underreported [5].

Recent study revealed that ethnic minorities had more frequently encountered conditions, including health access and health affordability, that are associated with an increased risk of COVID-19 than the general populations [6]. Moreover, ethnic minorities also had higher risk of getting depression, psychosocial stress, suicidal thoughts during the COVID-19 pandemic [7]. Social network of ethnic minority was more disrupted by COVID-19 pandemic than the general populations and interpersonal interaction among the ethnic minority groups was greatly affected by the containment measures [8].

Among ethnic minorities, women usually have different gender and social roles than men. During the pandemic, many ethnic minority women had to fulfill their caregiving roles and financially support their family simultaneously. These roles and responsibilities brought extra burden and made them more disadvantaged [9]. A study found that black women in the United States encountered extra problems including livelihood, economic burden, and psychological distress during COVID-19 and the impact on them was greater than on other ethnic groups [10]. Another study showed that South Asian (SA) women in Hong Kong were more disengaged from the healthcare system than Chinese women due to time constraint, financial burden, and limited healthcare access [11].

### General ethnic minorities situation in Hong Kong

Hong Kong is a generally ethnically homogenous city with about 92% Chinese. In 2016, there was a total of 584,383 ethnic minorities, constituting around 8% of the total population. Among the ethnic minorities who are not foreign domestic workers (FDW), SAs took up the largest proportion of 30.6% followed by the Mixed-race population (23%) and the Whites (21.9%) according to the Census and Statistics Department (C&SD) [12].

Average domestic household size of ethnic minorities was three, which was higher than that of the overall Hong Kong population (2.8 persons). Generally, SAs had larger families and had more children than the general population. For instance, Pakistanis and Nepalese had a bigger household size of 3.9 persons and 3.2 persons respectively, and more than one-fourth of Pakistani households had three children or more [12]. Regarding education attainment, lower proportion of SAs, including Pakistanis and Nepalese, and Southeast Asians (SEAs), including Thais and Indonesians, had attained post-secondary education while Whites, Japanese, and Koreans tended to be more educated. These less educated SAs and SEAs were mainly engaged in grassroots occupations and earned less [12]. The socioeconomic status of ethnic minorities varied across diverse ethnic groups. The median monthly employment income of the overall Hong Kong population was $15,500 HKD (i.e., around $1997 USD in 2016), while that of the White, Mixed-race, and SA populations were $18,000 HKD, $17,500 HKD and $15,000 HKD (i.e., around $2320 USD, $2255 USD and $1933 USD in 2016) respectively. Furthermore, the overall poverty rate of SAs was 23.0%, which was also higher than that of the overall Hong Kong populations (14.7%) in 2016 [12].

### Ethnic minorities situation during COVID-19 pandemic in Hong Kong

The Hong Kong Special Administrative Region of China (HKSAR) government announced the first COVID-19 infected case on January 23, 2020. There were 787,142 cases and 486,775 cases being tested positive by nucleic acid tests and rapid antigen tests respectively with 9419 deaths by July 11, 2022 [13]. Having experienced Severe Acute Respiratory Syndrome (SARS) epidemic in 2003, the HKSAR government and the community were vigilant, and reacted quickly in the early phase of the COVID-19 pandemic in 2020 [14]. While incidence of COVID-19 in Hong Kong was relatively low compared with other regions across the world, a number of studies showed that the economic situation and mental health of the general population were still severely influenced by the COVID-19 pandemic [15, 16].

According to the epidemiological data of COVID-19, ethnic minorities in Hong Kong do not have significantly higher infection rate and mortality rate than the general populations. However, they still suffered disproportionately during the pandemic. A recent study showed that SAs in Hong Kong possessed relatively poorer knowledge towards COVID-19 and they expressed certain misconceptions on prevention of COVID-19 infection [14]. Cultural background and religion were other important factors affecting their health seeking behavior [17].

Moreover, among the ethnic minority, women tend to be even more affected. Qualitative research found that SA women faced several difficulties, such as language barrier, unequal status of women, and limited health insurance in accessing healthcare in Hong Kong during normal days [11]. It has also been previously found that members of SA family with the least effective bargaining power often had to bear the brunt of the difficulties under external stress [18]; these members are usually, albeit not inevitably, women.

Excluding FDWs, SAs and SEAs account for the two largest ethnic groups of ethnic minorities in Hong Kong and have a higher overall poverty rate than other ethnic minorities. Therefore, in this study, we focused on SA and SEA minority women. Moreover, since existing literature about ethnic minorities situation during COVID-19 were mainly studies from western countries and regions with relatively high infection rate, our present study conducted in a context with relatively low incidence rate can fill this gap.

This study aimed at exploring the lived experiences and challenges encountered by South and Southeast Asian women in Hong Kong during the COVID-19 pandemic. By having better understanding of these challenges and the social determinants of health inequalities they encountered, our study findings may have important implications in the design and implementation of public health policies and strategies for any potential pandemic in the future.

## Methods

This qualitative study adopted in-depth semi-structured interviews with SA and SEA minority women in Hong Kong. The research team consisted of both male and female researchers. This method provides more comprehensive and in-depth understanding for human behavior and social phenomenon [19]. The result of qualitative study reveals multiple subjective views of individuals and the contextual background of participants which are usually overlooked in quantitative research [20].

### Sampling

This study employed purposeful sampling to select SA and SEA minority women who were residing in Hong Kong, aged over 18 years, and able to speak English or Cantonese. Participants were purposively sampled to have greater variation in ethnicity, education level, employment status, household size, and housing type. Working-age women are usually the major caretaker and occasionally breadwinner in their respective family. They tend to be more severely impacted during COVID-19 outbreak and might possess more insights into the issues at hand than others. Thus, working-age women were recruited. This sampling method aimed at selecting information-rich cases to reach data saturation [21]. The sampling process was reviewed continuously in order to achieve more diversity with recruitments [22].

### Data collection

Data were collected through individual interviews conducted by two male researchers with PhD qualifications and trained qualitative skills. The researchers first contacted social workers working in local non-governmental organizations (NGOs) which served ethnic minority. The social workers identified potential participants and referred them to researches after gaining participants’ verbal consent. Subsequently, the researchers explained the research objectives and interview process to the participants and conducted the one-on-one face-to-face interviews in community centers or via online video call (zoom).

A semi-structured interview guide was developed based on previous literature in order to study the impact of COVID-19 pandemic on the interviewees. It included open questions about participants’ physical and mental health conditions, economic situation and social interaction. Other open-ended questions were also asked to understand the daily life experience of participants amid the COVID-19 pandemic. Their demographic characteristics (including sex, age, marital status, and ethnicity) as well as COVID-19 infection and testing status were captured by a short survey.

Interviews were conducted in English or Cantonese, depending on the preference of interviewees. Each interview lasted for around 45 to 60 min, and was audio-recorded for face-to-face interviews or video-recorded for online video call interviews. All records were transcribed verbatim, and the Chinese texts were translated into English. Field notes were also made during the interviews. The interviews were carried out between September 2020 and March 2021.

### Data analysis

The written text transcribed from the recordings was inputted into NVivo, version 12, for analysis. Thematic analysis approach was employed and guided by Miles and Huberman’s suggestions [23] on qualitative analysis. To ensure the validity and credibility of our findings, the transcript was read several times by researchers to gain an overall understanding of meanings. The researchers then conducted open coding, summarized and extracted meaningful wordings. Codes were then further summarized into meaningful themes, clusters, and categories. Different opinions on the coding and themes were resolved by discussion within the research group. Credibility was established by investigator triangulation (i.e. peer debriefing) and data triangulation (i.e. checking data in field notes). Transferability was achieved by considering the characteristics and experience of participants through in-depth interviews [24].

## Results

### Basic description of participants

Ten SA and SEA minority women aged from 22 to 51 years were recruited. Their ethnicities include Indian, Filipino, Pakistani, Nepalese and Indonesian. Their education level ranged from junior school to degree or above. Six of them were homemakers while four of them were full-time employees. They lived in different types of housing, including public rental housing, private rental housing, and privately owned housing. Detailed background information of participants can be found in Table 1. No participant requested to drop out during or after the interview. Data saturation was achieved among these participants.

**Table 1:** Basic demographic information of participants

Participant	Age	Ethnicity	Education	Marital Status	Housing type	No. of family members	Employment Status	Monthly household Income (HKD/USD)	Language used in interview
A	51	Indian	Degree or above	Separated	Private rental housing	1	Full-time employee	35,000/4495	English
B	42	Philippine	Degree or above	Married	Private rental housing	6	Full-time employee	22,000/2825	English
C	32	Indian	Sub-degree	Married	Public Rental Housing	3	Homemaker	30,000/3853	English
D	43	Pakistani	Junior School	Married	Public Rental Housing	6	Homemaker	0	English & Cantonese
E	22	Nepalese	Sub-degree	Married	Private rental housing	4	Homemaker	23,000/2954	English
F	40	Indonesia	Lower secondary	Married	Private rental housing	6	Homemaker	21,000/2697	English
G	41	Nepalese	Upper secondary	Married	Privately owned housing	4	Homemaker	30,000/3853	English
H	43	Indian	Upper secondary	Married	Public Rental Housing	5	Homemaker	20,000/2568	English
I	23	Pakistani	Upper secondary	Single	Public Rental Housing	5	Full-time employee	25,000/3211	English
J	33	Nepalese	Sub-degree	Married	Private rental housing	2	Full-time employee	40,000/5137	Cantonese

In summary, although Hong Kong has a relatively low incidence rate of COVID-19 across the globe, ethnic minorities living in Hong Kong still face numerous physical, mental, economic, social, cultural, and religious challenges during the pandemic. Here we present the main findings by themes emerged from the qualitative interviews.

### Theme 1 family culture and gender role

During the interview, it was revealed that the role of married women in a family among SAs and SEAs living in Hong Kong is mainly to perform domestic hygiene, cooking and childcare. The burden of domestic hygiene increased disproportionately during the pandemic as ethnic minority women have to perform more frequent cleaning and disinfection. At the same time, they had to purchase masks and sanitizers in addition to other essential household products for the family. This brought extra burden to them especially during the beginning of COVID-19 outbreak when many essential products were in shortage in the local market:

*My husband is a bit of a traditional man who does not really like to perform house duties or cleaning. Now that we have to disinfect everything, he still does not care about his own clothes, so I have to handle them for him. [Before COVID-19], I used to clean the toilet at home two to three times per week. Now I have to disinfect it everyday. There are many cleaning tasks now. At the same time, I have to go to work, and cook for my family. My burden has in fact increased tremendously. Also, I have to buy masks, and do everything by myself, since my husband does not really care much. I think the overall workload of women of ethnic minorities have increased. I saw many similar cases. (Ms J)*

Meanwhile, SA and SEA women have to take care of their children and follow up on their academic performance. They encountered additional challenges on online schooling at home due to inadequate apparatus, including computer, headphone and Wi-Fi access, and limited space at home. They expressed additional concern when their children were distracted during online schooling, leading to poor academic performance. It is worth noting that some participants understand limited Chinese or English themselves and it was therefore difficult for them to understand and follow up on the learning progress of their children:

*I have four kids. One of my daughters is just ignoring the homework. I think it’s hard for her for the online class because she likes to do many things. She easily gets distracted. If the environment is different, …so she is not in the school, so just at home so I do easier accept [I would accept her behavior with more ease (sic)]. Suppose she will fail the S1 [author: secondary grade one], but since the situation getting worse, the teacher, her teacher, promoted her in considering [the] situation. My four kids have online teaching together using the headphone. One school provides the desktop for my other one kid, and then [for] the other one, I was forced [made (sic)] to buy outside because our computer [was] very old, it don’t have camera and speaker, so it is not good for he [him (sic)] to use. So I find other way, cheaper, went to buy. And [for] the two [other (sic)] teenagers, the school provides [for] them. But they will return it if something happens. [As for] the chromebook, we need to pay for it. (Ms B)*

*Before the Covid, the kids [were] going to school, right? So after the Covid, the kids [were] not going [to] school. But my time [to] wake up early [in the] morning are the same, 5.30 or 6am I wake up, because I need to make breakfast for my husband and make some food for my husband bringing for lunch [so that he could bring lunch (sic)] for his working. So, usually the kids will wake up and go to toilet, brush teeth, breakfast, and 8.15am will start in the Zoom… until 12.59pm, so almost 1pm… I need to [do] cooking, cleaning the house, hanging the clothes, busy like that, because I have four kids. All are boys and different class, so P3, P2, P1 [author: primary grade three, two, and one]. So it is very difficult for me … (Ms F)*

Furthermore, some SA and SEA minority women spent considerable time and effort in their own employment besides fulfilling the traditional gender role in their family. These extraordinary physical and mental demands create additional challenges for SA and SEA women in Hong Kong.

### Theme 2 mental and financial support of family members living outside Hong Kong

Families and friends of many ethnic minorities living in Hong Kong are still in their home countries. Some participants expressed their concern and worry of their relatives living in their home countries, especially those in areas with heavy COVID-19 caseload:

*My parents and my siblings are in India and my daughter studies in US, so I am in Hong Kong, so one of my hardest [it is especially hard for me (sic)] with my mother because she is 75 years old in India, and situation in India is not good, right? So one piece of my heart is already stucking [sticking close to (sic)] India’ news, … what’s happening [there], [and] talking to my mom. Another part of my heart is in US because people in United States, they are so … political, so many [much (sic)] social life. So, it is like that if something happens to them even though I can’t travel, I cannot go to see them. If my kid has some health issue, I cannot travel. If my mom has some problem, I cannot travel to India, right? Besides worrying for my family members, I have to be patient and taking care of myself as well. (Ms A)*

*[My husband] keeps on thinking, [what if] like any father or mother from my side or his side is [being tested] positive, then how [can] they get cured? Which hospital? And there are no vaccine [in] hospitals. Like you running [towards] this hospital [or] that hospital, [but] no vaccine. So situation there is worse. So that’s the problem. We are so confused. Stay here, the expenses are killing [us], [we] can’t even think [of going] back to India. (Ms C)*

In addition to mental burden, some ethnic minorities have to financially support their families living in their home countries during the pandemic. This added extra demand on the already challenging financial status of ethnic minorities in Hong Kong:

*Since the pandemic situation in Nepal is quite serious, all my families in Nepal lost their jobs. So, I have to support them by transferring some money to them. The amount is not much, but I tried my best to help them. That’s why I encountered some financial difficulties to support the demands not only in Hong Kong but also in Nepal. (Ms J)*

### Theme 3 economic activity and employment

From July 2020 to January 2021, Hong Kong implemented stringent public health measures, and many commercial and economic activities were severely impacted. People working in various industries were hit hard, and many people, including ethnic minorities, became unemployed or underemployed as a result:

*He [my husband] is working in construction, right? And then actually before Covid, his job is good, had overtime, something like that. But after Covid, actually the boss said “no more job.” He said like this, but because my husband and boss, the [their (sic)] relationship is good, long time already, maybe almost 7 or 8 years, so my husband talking [said, (sic)] “Please help, if you [do] not give me the job, how can I give my kids and my wife [food for] eating?” [He] said [it] like that. “It is okay you decrease the salary, but at least we work” he said like that. So now my husband still works, but sometimes no work. If [he] finished job, then no work … the boss will call another friend “Have job or not for him?” He said like that. Sometimes he comes back early so at least he has job, right? Even they say now is little bit [there is only a few jobs (sic)], but it is okay, [since] at least he still has job. (Ms F)*

When ethnic minorities tried to seek job in the market, they encountered a variety of difficulties due to their race and language barrier. This disparity in job opportunities was amplified during COVID-19 outbreak:

*One time, my friend, she went to an interview and I was with her. So first of all, the employer asked her if “you are from like which country” so she said she is from Pakistan. Then they refused to give her a job because they said there are so many [COVID] cases from Pakistan, so we are not hiring anyone from different countries, any EMs. So I guess it is discrimination. (Ms I)*

*My husband has been unemployed for six months. This affects our income…He works in construction, so it is difficult to find a job……He does not know Chinese. If he finds a job in construction, he has to find one with his ethnic minority friends. He cannot work in Chinese companies because of language problem. (Ms J).*

Moreover, being unemployed and unable to financially support the family caused extra stress on ethnic minorities, especially the men in SA and SEA family, who traditionally were regarded as the head of the household:

*I have been... very depression [depressed (sic)]. Because there was a lot of things...because my husband, he is also looking for a job. Because [for] the man, [if] he doesn’t have a job, then he has to stay at home. He always has negative thoughts. They [the ethnic minority families] have some quarrel between the families and between husband and wife. He is not happy because he thinks that he has to earn for his family, support his family. So, if one person, specially a man, he don’t have any job to do, … the problem is within ... it creates [the problem is created from (sic)] within a family. It disturbs our life… family lives. (Ms D)*

### Theme 4 information access

Due to language barrier, not all ethnic minorities are able to comprehend the latest local pandemic situation, understand the relevant public health measures thoroughly, and follow the measures accordingly. By misunderstanding and misinterpreting the information, “fake news” might have spread around the community:

*The first thing, because of the language problem, he just believes in false news. For example, if someone shared something like, tomorrow it’s gonna be everything would be shut down… they can easily believe in fake news. One of the major difficulties is that…because of the language problem, they do not understand. So the fake news just continued to spread to other members [in the community]. (Ms I)*

Furthermore, at the beginning of the pandemic in early 2020, some local information, including the sales of masks and sanitizers, was only published online in Cantonese. It is challenging for ethnic minorities to access such information and purchase these essential products, especially when these products were in severe shortage in the local and global market.

*I remember at the beginning, I just bought two boxes of masks. My colleague bought them for me. When they were finished, I felt scared, scared about how to buy and where to buy them. It’s expensive outside. Even if you could pay, you can’t buy it [author: since they were in shortage]. I was scared at the time. Luckily I found my Chinese colleague who helped order them for me. But I knew many people who did not have Chinese friends, [so] they had no mask. And they usually didn’t know what was happening at the moment, or how many confirmed cases, … this type of information… (Ms J)*

### Theme 5 cultural sensitivity, social network and religious support

Participants expressed that they felt being disrespected or discriminated on many occasions because of their ethnicity during the pandemic. They raised real-life examples of being physically or verbally offended by others:

*I always notice like the lift we passed, on the elevator lift, some people, they, tried to avoid us, like I don’t know why. Like “go away, go away!” They [were] scared that we [were] going to spread [the disease]. Hahahaha. I was thinking we are not carrying the coronavirus with us. Many times I feel that people are trying to like being away, we are not very close to them but in the lift, definitely kids sometimes hold hand like this [author: gesture of covering mouth and nose] , so they [the ethnic minotrity people] are very angry. (Ms C)*

HKSAR government implemented stringent public health measures to lockdown certain districts, buildings or facilities in order to carry out mandatory surveillance and testing. According to the C&SD [25], Yau Tsim Mong district had the highest proportion (i.e., 9.1%) of ethnic minorities out of all 18 districts in Hong Kong. Lockdown in Yau Tsim Mong left ethnic minority communities feeling vulnerable, targeted and shunned. They expressed concerns over the speech of a senior government health officer that ethnic minorities were at higher risk of spreading the virus because they had many family gatherings and were likely to share foods, smoke, drink alcohol, and chat with each other without masks:

*…especially during lockdown in Jordan, someone from the Health Department said something that affected us. Nepalese have many facebook groups. They shared that someone talked about us. Many people were not happy and angry. I have heard that when one Nepalese woman came out from the MTR, a Chinese blamed her and spitted on her. She was very frightened and did not report to the police at that time. She asked for advice in the group. Finally the district counselor followed up on the case. (Ms J)*

*They [someone from the Centre for Health Protection of the Government] said that we sat and drank beers in the park and did not wear masks. It seems that we created the problem. When there were lockdowns in other areas, they did not use ethnicity to describe or discriminate. But during lockdown in Jordan, they said that our communities were dirty and we lived in small subdivided flats. It seems that our lifestyle was very disgusting and unhygienic. (Ms J)*

From July 2020 to January 2021, Hong Kong Government implemented stringent social gathering restriction; thus, the ethnic minorities experienced difficult moments with limited social support:

*At that time [when the Government implemented stringent social gathering restriction], there was more home office. [I] cannot see people all the time, so [I] became quite distressed at that time. It’s not depression but stress, because human needs to socialize. [I] became unhappy, very tired and cannot sleep. (Ms J)*

Also, having not gone to mosque imposed mental burden on ethnic minorities with religious belief because they gained limited religious and social support:

*…stress relieve. So going to temple, going out with my friends for dinner or lunch or seeing each other. Sometimes we just go for coffee for 2-3 hours. So sharing your feeling, seeing someone face to face. You can just imagine, in the conversation, you can feel the energy between you and me. Just feel [imagine (sic)] the same conversation on the Zoom, would you feel really the same? Definitely not, right? So that is the difference… We feel the difference with electronic, we cannot see face to face. So the same [situation is] impacting your life, your relationship. (Ms A)*

### Theme 6 government and non-governmental organization support

Some ethnic minorities mentioned that government social services were very important to their members, since they were not able to speak the local language:

*First of all, I’m worried about Chinese before, first time we came, we [were] a bit...struggled [we were struggling a bit (sic)], because at that time there was no social service. We didn’t have this kind of knowledge...about ethnic minorities, [that] they also had a social service [that they were also entitled to social service (sic)]. There are so many social service centers that can help, before it’s a bit hard time, because [of] language problem, and difficult to get jobs…because more knowledge [were] needed… especially we need government facilities because the, yea...because the government are doing all, I mean not only ethnic minorities, all, the government of Hong Kong people. (Ms H)*

In addition to seeking support from government services, ethnic minorities were also likely to utilize resources provided by different NGOs during the COVID-19 pandemic. For example, they obtained masks and hand sanitizers from these NGOs. They would also help to distribute the products they received from the NGOs to other members within their community:

*… I went to all the NGOs, ‘cause mostly I have joined many NGOs, like community house, ah sorry, the Christian Action, Home Centre, Seeds for Chinese, I know most of the community, we attend the class there, and they always help us to distribute the masks. For masks, I really want to help the, like hand sanitizer and for home safety advances, … the NGOs help a lot to distribute the masks and things like that. So till now… Still going on, sometimes school also, some schools also distribute the mask, and hand sanitizer, like that. (Ms G)*

Nevertheless, their experience with the government was not all positive. HKSAR government implemented strategies to isolate close contacts of confirmed positive cases or inbound travelers in designated quarantine facilities for 14–21 days until they were tested negative. However, support for ethnic minorities was considered inadequate and inappropriate during the quarantine period since they had limited food choice:

*Quarantined in the government quarantine center which was at Fo Tan…We were literally dying for food. My child was not eating at all because at that time, you know, … they were giving two types of big boxes of rice and they don’t eat rice…The food problem was dangerous for us because I didn’t have snack, I didn’t have food and they didn’t allow me to buy a bread also. I said, “please my friend, at least give me some bread,” [And they said,]“We cannot allow you.” Nothing outside can come inside. They [were] all Chinese. (Ms C)*

## Discussion

During the pandemic, ethnic minorities in Hong Kong experienced various challenges due to their unique family culture and bonding, insecure employment status and economic activity, as well as language and information access barrier.

In this study, SA and SEA minority women reported increased responsibilities for domestic duties and childcare during COVID-19 pandemic. This is consistent with previous literature that women were given more domestic responsibilities while men predominantly had non-domestic roles in traditional societies [26]. Traditional thoughts of some ethnic minorities are that men are responsible for earning money and supporting the whole family financially. When a man becomes unemployed, he no longer fulfills his traditional role and this may create tension within the family. Furthermore, the close bonding and familial tie between the ethnic minorities in Hong Kong and their relatives in home countries is consistent with Ballard [18] who found that most South Asian migrants have made great efforts to sustain the unity of their families, and their familial obligations did not go away even after migration.

According to the C&SD [27], the overall unemployment rate peaked at 7.2% during the period from December 2020 to February 2021 in Hong Kong. However, it is very likely that ethnic minorities were disproportionately impacted in this unemployment crisis because of the industries in which the majority of them worked. According to the C&SD [25], 74.4% of the working ethnic minorities were engaged in elementary occupations. The NGO Hong Kong Unison [28] found that a large proportion of SA workers worked as security guards, construction workers, clerical workers, and delivery workers. Among the unemployed labor forces in the market during the pandemic, construction industry accounted for the highest unemployment rate at 8.1%, followed by retail, accommodation and food services industry at 7.6% [27].

Furthermore, ethnic minorities reported extra difficulty when they sought for job opportunity in the market due to language barrier, or education level [28]. It is worth noting that only 44.2% of ethnic minorities reported English was the language most commonly spoken at home followed by Cantonese (31.7%) [28]. Even if some ethnic minorities can speak Cantonese Chinese and/or English, they may not be able to read or write the language. During the pandemic, local government releases COVID-19 related information and announcement mainly in two official languages: Cantonese Chinese and English; thus, some ethnic minorities were not able to understand local pandemic situation and follow the corresponding infection control measures accordingly. This is consistent with a recent study which reported that SAs in Hong Kong might possess insufficient knowledge about COVID-19 as they were not able to comprehend the information as disseminated by the government [14]. Moreover, due to misunderstanding of the information, “fake news” could easily be circulated in the ethnic minority communities. This poses threat to the implementation of the public health strategies by reducing their efficiency and effectiveness.

On top of all the challenges mentioned above, ethnic minorities also faced alleged discrimination and/or verbal offense during the pandemic, because of their physical resemblance to the people in countries being hit hard by COVID-19, not because of any evidence of them being the carrier of the virus. This phenomenon is consistent with various reports from the US indicating events of discrimination and heightened exclusion against minorities during the COVID-19 crisis [29]. Similar phenomenon against migrant workers was also reported in Singapore during the pandemic [30]. It is even less helpful if the stigmatized speech against ethnic minorities came from government official, who should have acted in the interest of all people of the population, regardless of race, ethnicity or socioeconomic position.

### Recommendations

Implementation of stringent but essential public health measures to control the spread of coronavirus is unavoidable during the COVID-19 pandemic. Nevertheless, it is equally important to carefully consider social determinants of health in addition to the direct physical impact of coronavirus when designing the corresponding public health strategies.

In this study, the issue of employment and job security is reiterated by the participants. It is burdensome for ethnic minorities to find suitable and stable jobs even before the COVID-19 outbreak. This underlying and persistent issue of job security is further amplified during the pandemic. Therefore, government departments responsible for labor and social affairs should bear responsibility to strengthen and enhance current employment services for ethnic minority job seekers. For example, sponsorship for on-job training and language training for ethnic minorities could be provided by the government. This has public health implication since it has been found that poorer mental health could be partly explained by people’s concerns over their livelihood and economic activity during the pandemic [31].

In addition, it is critical to acknowledge, respect and address the culturally specific needs of the ethnic minorities, which may include but are not limited to their dietary preference, cultural custom, and religious beliefs. For example, adequate and appropriate food of choice could be provided to ethnic minorities during mandatory quarantine. Additionally, public education could be enhanced for local and ethnic minority communities in order to promote racial inclusion.

### Limitations

First, this study design is qualitative in nature. In other words, the results cannot statistically represent the views of all ethnic minorities in Hong Kong. Nevertheless, the purposively selected interviewees were all working-age women, who were also major caretakers and possible breadwinners in their respective families. They might possess more insights into the issues at hand than other ethnic minorities as they tend to be more severely impacted by the pandemic due to the intersectionality of ethnicity, socioeconomic position, occupational status, education, and gender. On the other hand, a more comprehensive picture could be drawn if adolescents and older ethnic minorities were included. Second, although the interviewers were well-trained while the interviewees were recruited from multiple sources, the results may be biased due to the use of semi-structured interview guide, the relatively low level of English proficiency of some participants, and voluntary participation. However, our interviewers clarified with the interviewees whether their words were correctly understood, whenever necessary, in order to minimize error in transcription and interpretation. Third, our study participants were predominantly SAs and SEAs, and other ethnic minorities including Africans, Americans and Europeans were not included in our study. Nevertheless, there were much fewer Africans in Hong Kong, and Americans and Europeans represent a more diverse group of ethnic minorities that consist mainly of expats of higher socioeconomic position. Additionally, ethnic minorities who do not speak either English or Chinese were not recruited in this study. While there is reason to believe that they were even more marginalized during the pandemic, they represent a sub-population of the ethnic minority who are even more elusive and invisible.

## Conclusions

Hong Kong can act as an exemplar setting of an Asian city with grossly homogeneous population that is mixed with diverse ethnic minorities. Even when the COVID-19 incidence rate is relatively low in Hong Kong, the pandemic made life even more challenging for a community already struggling with language barriers, financial woes, and discrimination, which in turn could also lead to greater health inequalities. Government should take a leading role, in cooperation with civil organizations, to implement public health policies and strategies that contain the virus while taking the social determinants of health inequalities into account.

## Data Availability

The datasets generated and/or analyzed during the current study are available from the corresponding author on reasonable request.

## Abbreviations

C&SD: Census and Statistics Department
FDW: Foreign domestic workers
HKSAR: Hong Kong Special Administrative Region of China
NGO: Non-governmental organization
SA: South Asian
SEA: South-east Asian
